# Investigating the Structure of Intelligence Using Latent Variable and Psychometric Network Modeling: A Commentary and Reanalysis

**DOI:** 10.3390/jintelligence9010008

**Published:** 2021-02-05

**Authors:** Christopher J. Schmank, Sara Anne Goring, Kristof Kovacs, Andrew R. A. Conway

**Affiliations:** 1 Department of Psychology, Claremont Graduate University, Claremont, CA 91711, USA; sara.goring@cgu.edu (S.A.G.); andrew.conway@cgu.edu (A.R.A.C.); 2 Institute of Psychology, ELTE Eotvos Lorand University, 1064 Budapest, Hungary; kovacs.kristof@ppk.elte.hu

**Keywords:** intelligence, psychometric network analysis, latent variable modeling, statistical modeling, WAIS-IV, theory compatibility

## Abstract

In a recent publication in the *Journal of Intelligence,* Dennis McFarland mischaracterized previous research using latent variable and psychometric network modeling to investigate the structure of intelligence. Misconceptions presented by McFarland are identified and discussed. We reiterate and clarify the goal of our previous research on network models, which is to improve compatibility between psychological theories and statistical models of intelligence. WAIS-IV data provided by McFarland were reanalyzed using latent variable and psychometric network modeling. The results are consistent with our previous study and show that a latent variable model and a network model both provide an adequate fit to the WAIS-IV. We therefore argue that model preference should be determined by theory compatibility. Theories of intelligence that posit a general mental ability (general intelligence) are compatible with latent variable models. More recent approaches, such as mutualism and process overlap theory, reject the notion of general mental ability and are therefore more compatible with network models, which depict the structure of intelligence as an interconnected network of cognitive processes sampled by a battery of tests. We emphasize the importance of compatibility between theories and models in scientific research on intelligence.

## 1. Introduction

In a recent issue of the *Journal of Intelligence*, McFarland (2020) compares traditional latent variable models of intelligence to more recent psychometric network models. In contrast to Kan et al. (2019) and Schmank et al. (2019), McFarland finds that latent variable models generally outperform network models. McFarland suggests that previous support for network models reported by Kan et al. (2019) and Schmank et al. (2019) is limited to analyses based on partial, rather than uncorrected, correlation matrices. If correct, this finding calls into question recent claims that psychometric network models provide unique support for theories of intelligence like mutualism and process overlap theory. 

We are grateful to McFarland for providing such a detailed comparison of latent variable and network models of intelligence. In many ways, it is representative of his program of research on intelligence, which managed to combine statistical rigor and theoretical impact. Indeed, it has forced us to reconsider both methodological and theoretical aspects of our recent work on psychometric network models of intelligence. It has also provided us with plenty of food for thought and ideas for future research. 

At the same time, unfortunately, we get the impression that McFarland misunderstood some methodological details, as well as the overall purpose of Kan et al. (2019) and Schmank et al. (2019). Kan et al. (2020) have already addressed McFarland’s misconceptions of Kan et al. (2019) and so we focus here on issues that pertain to Schmank et al. (2019). We also report an analysis of Wechsler Adult Intelligence Scale–Fourth Edition (WAIS-IV) data generously provided by McFarland. We conducted the same exact statistical procedures reported in Schmank et al. (2019). To preview, the results are consistent with Schmank et al. (2019) and Kan et al. (2019) and illustrate that network models can account for the psychometric structure of intelligence and should therefore be considered as a viable alternative to latent variable models. For many investigators, network models are an attractive alternative because they are more compatible with recent theories of intelligence like mutualism (Van Der Maas et al. 2006) and process overlap theory (Kovacs and Conway 2016). We argue that compatibility between theories and models of intelligence can, and should, guide model selection. 

We review here two general concerns with the discussion of our previous research by McFarland (2020). First, McFarland states, “[t]he rationale for the use of partial correlations is that one can rule out the relationship between any pair of variables as being due to the other variables in the analysis and thus more readily infer causation” (p. 1). We agree that partial correlations can be used for this purpose; however, this was not the rationale behind the Schmank et al. (2019) study. Psychometric network modeling offers an alternative approach to investigations of the underlying structure of individual differences in cognitive abilities (i.e., intelligence), without the assumptions that come with reflective latent variable models (Conway et al. 2021; see also Goring et al. 2019). According to the view of intelligence endorsed by Schmank et al. (2019), process overlap theory (Kovacs and Conway 2016), there is no need to assume that (all or some of) the factors in higher-order factor models represent common causes of individual differences in test performance. It is in this sense that process overlap theory fits with the network approach, which also does not assume the presence of reflective latent variables. 

Second, McFarland (2020) states “[b]oth Kan et al. (2019) and Schmank et al. (2019) compared model fit indices for network models with those for traditional latent variable models” (p. 1). He then goes on to state “[o]ne problem in attempting to compare network models with traditional latent variable models […] is that the two deal with different aspects of test correlations” (p. 2). The first statement is inconsistent with Schmank et al. (2019): “[d]irect model comparisons were not conducted […] we caution readers from making direct comparisons based on the presented model fit indices” (p. 7). To be clear, these two kinds of models can be compared in principle, namely when they are both confirmatory, but this was not the case here. The second statement is also inaccurate. The data for all latent variable and psychometric network modeling analyses were uncorrected correlation matrices. 

To be sure, we reviewed our R scripts (https://osf.io/3wpcm/) but we found no direct comparisons of network models and latent variable models or partial correlations matrices. However, in our original pre-print of the Schmank et al. (2019) publication (https://osf.io/f9d2v/), we did report a direct comparison of the models. We therefore assume that the pre-print was the source of the confusion. To be clear, that pre-print was an unpublished draft and was later revised. The direct comparison between the model fit of latent variable and psychometric network models was not included in Schmank et al. (2019).

### Commentary Reanalysis

The remainder of this commentary provides a statistical reanalysis of the uncorrected WAIS-IV correlation matrices used by McFarland (2020). McFarland provided these correlation matrices to our research group and we then conducted the same set of analyses reported by Schmank et al. (2019). Thus, these correlation matrices were used as input for reflective latent variable modeling and psychometric network modeling, respectively. First, confirmatory factor analyses were conducted to determine the underlying measurement model of the WAIS-IV data assuming a correlated latent variable model. Next, we assessed the Cattell–Horn–Carrol model (Carroll 1993; Cattell 1943; Horn and Cattell 1966)—a reflective, higher-order model of intelligence—using an additional confirmatory factor analysis. Finally, psychometric network modeling was employed as an alternative method to traditional latent variable modeling techniques. For all psychometric models considered we provide model fit indices.

## 2. Materials and Methods 

### 2.1. Participants and Measures

The WAIS-IV correlation matrices provided by McFarland (2020) were based on three groups of participants (which we refer to here as younger, middle, and older). The age range of the younger group was 16–19 (*n* = 400); the age range of the middle group was 20–54 (*n* = 1000); the age range of the older group was 55–69 (*n* = 400). The three uncorrected correlation matrices were used as input for all confirmatory latent variable and exploratory psychometric network models. The analyses were conducted by following the Statistical Procedure outlined in Schmank et al. (2019).

The WAIS-IV consists of 15 subtests described as Information, Vocabulary, Comparisons, Similarities, Picture Completion, Block Design, Figure Weights, Matrix Reasoning, Visual Puzzles, Arithmetic, Digit Span, Letter–Number Sequencing, Cancellation, Coding, and Symbol Search. Information about these measures can be found in the Technical and Interpretative Manual (Wechsler 2008).

Two latent variable models were tested: a correlated four-factor model (Model 1), and a higher-order reflective *g* four-factor model (Model 2). Model 2 is based on the Cattell–Horn–Carroll model (Carroll 1993; Cattell 1943; Horn and Cattell 1966). Model fit indices were examined to compare Models 1 and 2 and to determine whether the same model fits equally well across groups when using the same modeling technique (i.e., psychometric network modeling or latent variable modeling).

### 2.2. Statistical Procedure and Analysis

#### 2.2.1. Confirmatory Factor Analyses

As an initial step, factor analyses were conducted to assess whether the provided correlation matrices demonstrated the same underlying structure (i.e., measurement model). The current project employed a measurement model with four latent variables representing crystallized intelligence, fluid reasoning, working memory, and processing speed. Additionally, a higher-order model was assessed by specifying a latent variable model with one superordinate second-order latent variable (representing g), and four subordinate first-order latent variables (crystallized intelligence, fluid reasoning, working memory, and processing speed). Latent variable models were conducted using lavaan (Rosseel 2012) and openMx (Neale et al. 2016) packages freely available in R (R Core Team 2013). The most parsimonious latent variable model is displayed in figures using visualization software freely available using Ωnyx (von Oertzen et al. 2015). For access to the R-scripts used in this project, see the following OSF project page: https://osf.io/kfujs/.

#### 2.2.2. Psychometric Network Analyses

Psychometric network analyses were conducted using the default estimation strategy provided by the qgraph package. For each psychometric network model conducted, 100 network models were generated with varying tuning parameter lambda values. Additionally, the ratio of minimum to maximum lambda values was set to 0.01 as specified by Epskamp et al. (2018). The final network was selected by the qgraph package by minimizing the Extended Bayesian Information Criteria utilizing the specified hyperparameter gamma that was set to 0.50. Psychometric network analyses were conducted using qgraph (Epskamp et al. 2012) and openMx (Neale et al. 2016) packages freely available in R. These network models were visualized using qgraph. Additionally, the average Layout () function within the qgraph package was used to make an average layout that can be beneficial for comparing the interconnectivity of each network model. It is important to reiterate that these packages were previously used in publications concerning the use of psychometric network analysis on cognitive ability measures (Kan et al. 2019; Schmank et al. 2019; Van Der Maas et al. 2017). Finally, the EGAnet (Golino and Epskamp 2017) package and EGA () function were used to assess the number of clusters (i.e., latent dimensions) and which observed variables belong to which cluster. 

#### 2.2.3. Approach to Model Fit 

The same approach to model fit evaluation was used in the current project as described in Schmank et al. (2019). Model fit will be deemed appropriate when (a) the ratio of model chi-square (χ^2^) to degrees of freedom is less than or equal to 3.00, (b) comparative fit indices (e.g., Comparative Fit Index (CFI) and Tucker–Lewis Index (TLI)) greater than or equal to 0.95, and (c) Root Mean Square Error of Approximation (RMSEA)^1^ values less than or equal to 0.06. Additionally, Akaike Information Criteria (AIC) and Bayesian Information Criteria (BIC) values can be used to compare models: smaller values indicate better fit. Furthermore, fit indices were extracted using functions within the lavaan, openMx, and qgraph packages which compare original covariance matrices to the implied covariance matrices generated by each latent variable and psychometric network model.

## 3. Results

The correlation matrices used in the current project are presented in Table 1, Table 2 and Table 3. Model fit indices are presented in Table 4, Table 5 and Table 6. Two sets of fit indices are reported because we estimated model fit using two R packages: lavaan and openMx. The packages differ with respect to model specification and estimation but the results here are identical. This was established and reported here to check the accuracy of our results and to facilitate reproducibility. 

**Figure 1:**
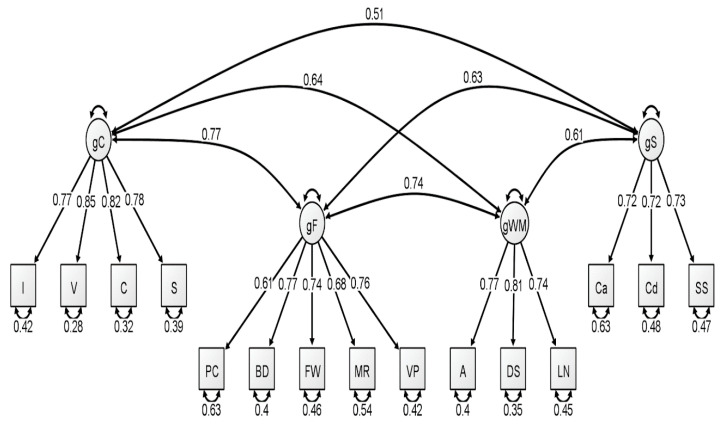
Correlated four-factor measurement model of the young group WAIS-IV correlation matrix. All values are standardized from the confirmatory factor analysis conducted using lavaan. Figure generated using Ωnyx. gC = crystallized intelligence; gF = fluid reasoning; gWM = working memory; gS = processing speed; BD = block design; S = similarities; DS = digit span; MR = matrix reasoning; V = vocabulary; A = arithmetic; SS = symbol search; VP = visual puzzles; I = information; Cd = coding; LN = letter–number sequencing; FW = figure weights; C = comparisons; Ca = cancellation; PC = picture completion.

**Figure 2:**
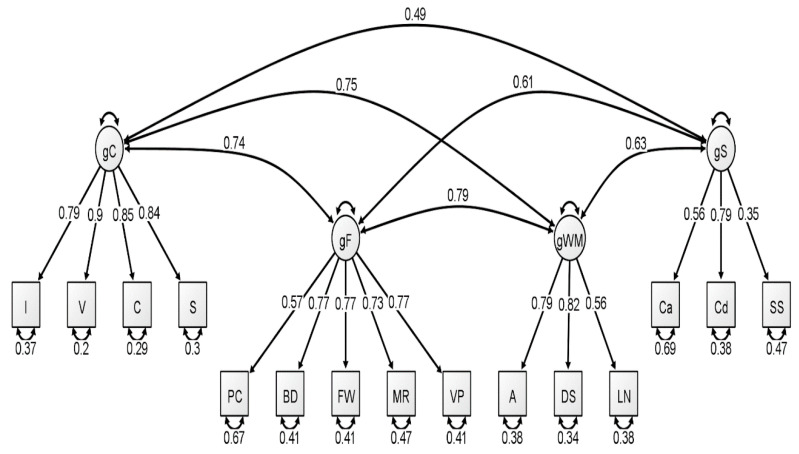
Correlated four-factor measurement model of the middle group WAIS-IV correlation matrix. All values are standardized from the confirmatory factor analysis conducted using lavaan. Figure generated using Ωnyx. gC = crystallized intelligence; gF = fluid reasoning; gWM = working memory; gS = processing speed; BD = block design; S = similarities; DS = digit span; MR = matrix reasoning; V = vocabulary; A = arithmetic; SS = symbol search; VP = visual puzzles; I = information; Cd = coding; LN = letter–number sequencing; FW = figure weights; C = comparisons; Ca = cancellation; PC = picture completion.

**Figure 3:**
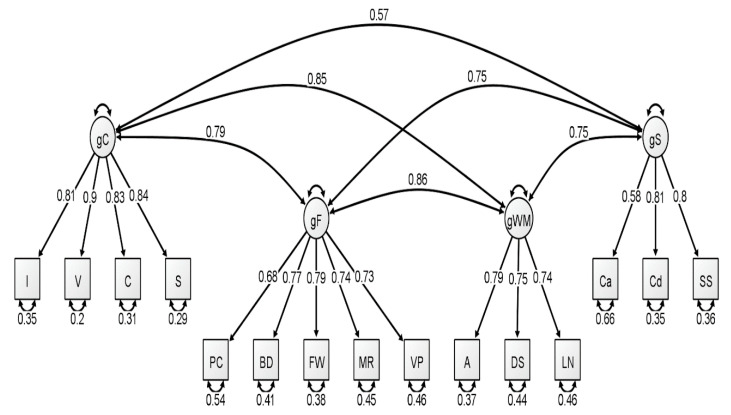
Correlated four-factor measurement model of the older group WAIS-IV correlation matrix. All values are standardized from the confirmatory factor analysis conducted using lavaan. Figure generated using Ωnyx. gC = crystallized intelligence; gF = fluid reasoning; gWM = working memory; gS = processing speed; BD = block design; S = similarities; DS = digit span; MR = matrix reasoning; V = vocabulary; A = arithmetic; SS = symbol search; VP = visual puzzles; I = information; Cd = coding; LN = letter–number sequencing; FW = figure weights; C = comparisons; Ca = cancellation; PC = picture completion.

With respect to the latent variable modeling analysis, the correlated four-factor model (Model 1) was preferred over the higher-order model (Model 2), based on model comparisons: Younger Group: Δχ^2^(2) = 5.56, *p* = 0.06; Middle Group: Δχ^2^(2) = 15.36, *p* < 0.001; Older Group: Δχ^2^(2) = 21.01, *p* < 0.001. For a visualization of these three models, see Figure 1, Figure 2 and Figure 3. In our original publication (Schmank et al. 2019), we presented data representative of the higher-order (g theory) model of intelligence, as this model tends to be the most well-known specification of latent variable models of intelligence. However, our findings after conducting these analyses on the McFarland data are inconsistent with a preference for the higher-order model of intelligence. Thus, when considering latent variable modeling, the three standardized WAIS-IV correlation matrices used by McFarland fit best when specifying a correlated, four-factor latent variable model.

Consistent with the results of Schmank et al. (2019), most fit indices indicated that the latent variable models provided an acceptable fit to the WAIS-IV data, with the exception of the chi-square test, which was nonsignificant for all latent variable models. However, the ratio of χ^2^ to degrees of freedom was less than 3.00 for three latent variable models: each model attributed to the younger group and for the higher-order model attributed to the older group. The reported RMSEA values for all latent variable models exceeded the conservative value used by the current authors to demonstrate appropriate model fit (all latent variable RMSEA values were 0.07). However, these RMSEA values would be adequate based on more liberal standards (see Browne and Cudeck 1993; Schermelleh-Engel et al. 2003). Additionally, comparative fit indices (i.e., CFI and TLI) demonstrated values in the acceptable range for all latent variable analyses. We also considered the quality of each measure by evaluating the standardized factor loadings in each model. The squared value of the standardized factor loading indicates the amount of variance in the measure that can be explained by the overarching latent construct. For the younger group, only two standardized loadings, specific to the fluid reasoning latent variable, failed to surpass the cutoff value of 0.70 that is typically used to indicate a quality measure (see Schmank et al. 2019). For the middle group, four standardized loadings failed to surpass the 0.70 cutoff value, specific to fluid reasoning, working memory, and processing speed. Finally, the oldest group demonstrated two standardized loadings that failed to reach 0.70, specific to the fluid reasoning and processing speed latent variables. 

With respect to the exploratory psychometric network modeling analysis, consistent with Schmank et al. (2019), the network models indicated excellent model fit across the majority of reported fit indices. First, only the psychometric network model for the middle group demonstrated a statistically significant χ^2^ value (*p* < 0.05); however, the value of the ratio between χ^2^ and degrees of freedom for each of these models was well below the 3.00 cutoff value determined prior to analyses. Finally, the comparative fit indices (i.e., CFI and TLI) demonstrated near perfect fit, while RMSEA values were well below the cutoff value specified. 

**Figure 4:**
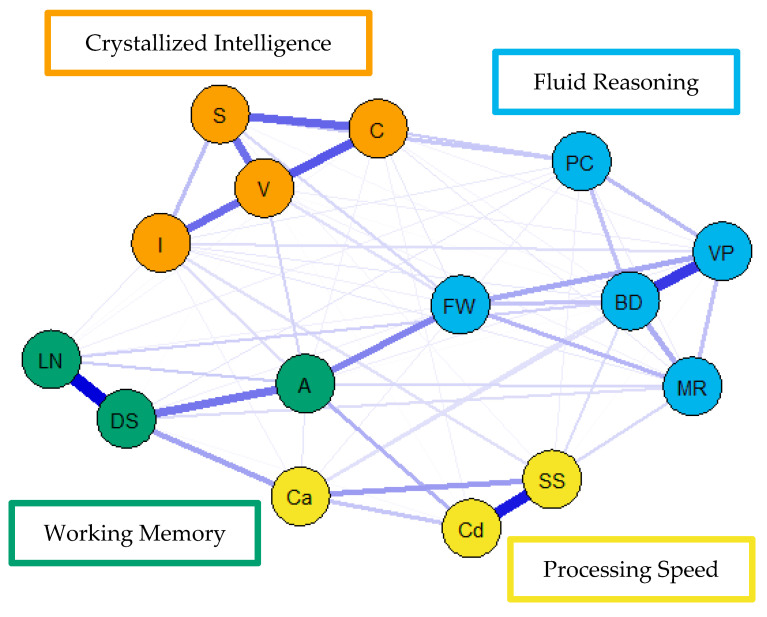
Weighted, undirected psychometric network model of the young group WAIS-IV correlation matrix. BD = block design; S = similarities; DS = digit span; MR = matrix reasoning; V = vocabulary; A = arithmetic; SS = symbol search; VP = visual puzzles; I = information; Cd = coding; LN = letter–number sequencing; FW = figure weights; C = comparisons; Ca = cancellation; PC = picture completion.

**Figure 5:**
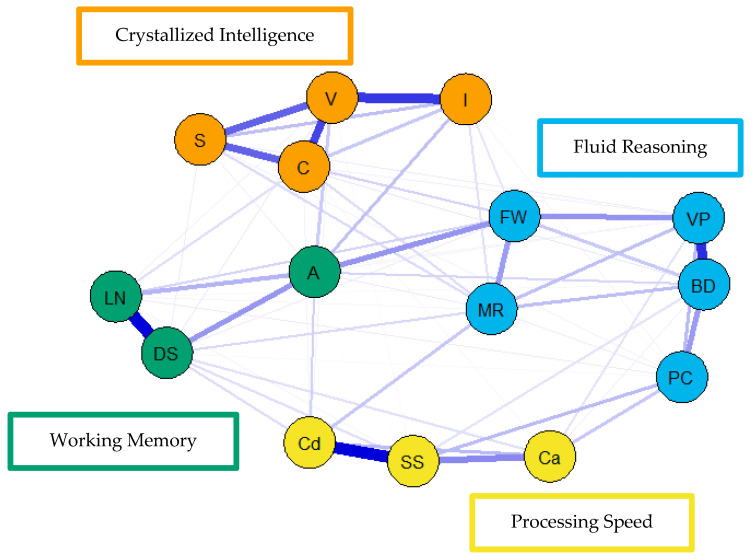
Weighted, undirected psychometric network model of the middle group WAIS-IV correlation matrix. BD = block design; S = similarities; DS = digit span; MR = matrix reasoning; V = vocabulary; A = arithmetic; SS = symbol search; VP = visual puzzles; I = information; Cd = coding; LN = letter–number sequencing; FW = figure weights; C = comparisons; Ca = cancellation; PC = picture completion.

**Figure 6:**
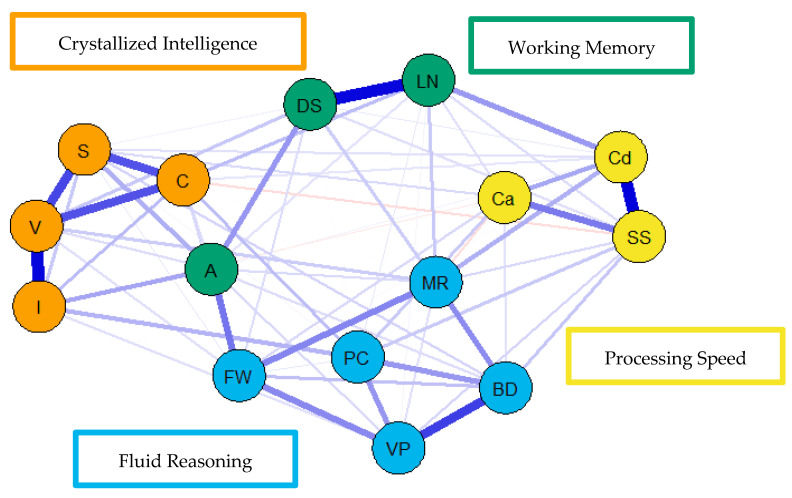
Weighted, undirected psychometric network model of the older group WAIS-IV correlation matrix. BD = block design; S = similarities; DS = digit span; MR = matrix reasoning; V = vocabulary; A = arithmetic; SS = symbol search; VP = visual puzzles; I = information; Cd = coding; LN = letter–number sequencing; FW = figure weights; C = comparisons; Ca = cancellation; PC = picture completion.

**Figure 7:**
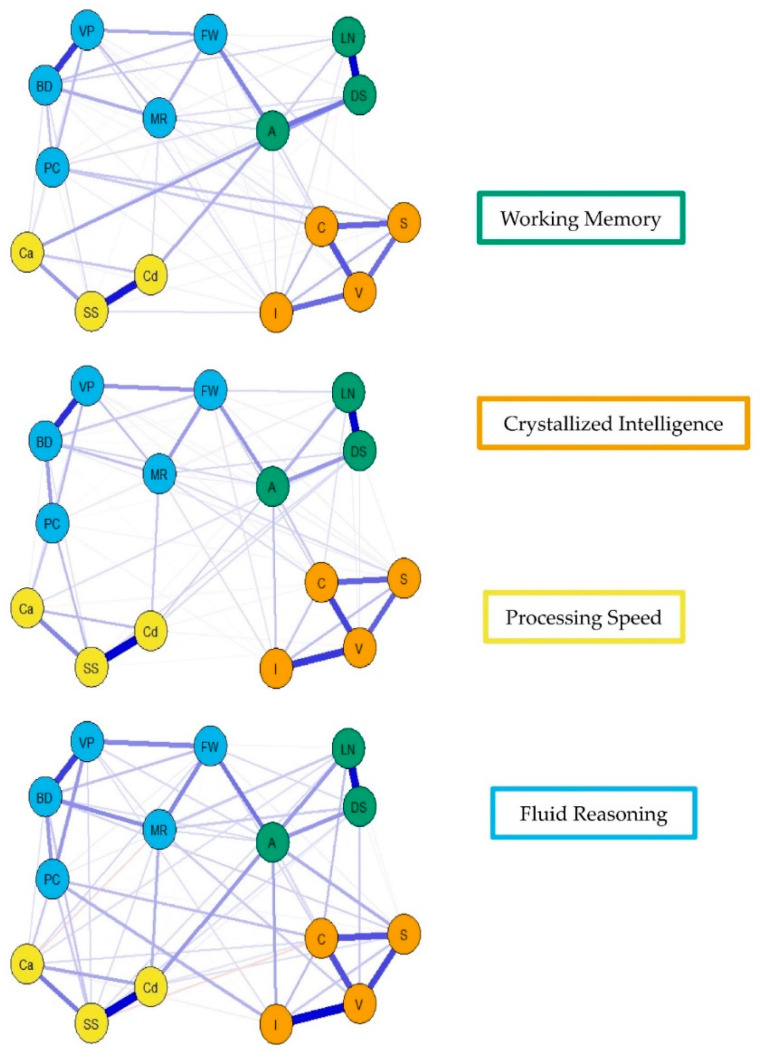
Youngest (**top**), middle (**middle**), and older (**bottom**) group WAIS-IV weighted, undirected psychometric network models with averaged layout.

For the visualization of the psychometric network models, see Figure 4, Figure 5 and Figure 6. First, nodes in the models have been colored to reflect the latent structure of the finalized measurement model. Second, the connections between nodes have been colored so that blue and red indicate positive and negative partial correlations, respectively. Third, the width of each connection between nodes represents the magnitude or size of the association or partial correlation estimated between each pair of cognitive task nodes. Additionally, the psychometric network models and their respective exploratory graph analyses revealed four clusters or latent dimensions. For the young (Figure 4), middle (Figure 5), and older groups (Figure 6) these four dimensions reflect crystallized intelligence, fluid reasoning, working memory, and processing speed. Interestingly, the Arithmetic (A) node in the older group network was explained by the crystallized intelligence dimension and not the working memory or fluid reasoning dimensions. Finally, three additional network models were generated that shared an averaged network layout (Figure 7).

## 4. Discussion

The current commentary addressed several inaccurate statements made by McFarland (2020). The commentary also provided an opportunity to further examine confirmatory latent variable models and exploratory psychometric network models of intelligence. Our general goal was to establish that psychometric network modeling can provide an alternative approach of the psychometric structure of test scores from the WAIS-IV like latent variable modeling. Furthermore, uncorrected correlation matrices of standardized WAIS-IV data provided by McFarland (2020) were submitted to the statistical procedures used by Schmank et al. (2019). 

The results are consistent with the findings presented in Kan et al. (2019) and Schmank et al. (2019). Based on the criteria provided by Schreiber et al. (2006), when confirmatory latent variable modeling and psychometric network modeling techniques were applied to the WAIS-IV correlation matrices provided by McFarland (2020), the resulting model fit indices were generally within what best practices and standards deem acceptable. From the perspective of model fit indices, we have corroborated the major findings presented by Kan et al. (2019); however, due to the exploratory nature of the psychometric network models and the confirmatory nature of the latent variable models, a direct comparison was not tenable as these types of analyses represent two separate stages of the psychometric research process, exploration, and confirmation. 

It is worth restating the main point presented in Schmank et al. (2019): that theories of intelligence like mutualism and process overlap theory (Kovacs and Conway 2016) are incompatible with reflective, higher-order latent variable models. By assuming process overlap theory, *g* is viewed as an emergent property or index and not some higher-order reflective factor. Process overlap theory is, however, compatible with psychometric network analysis. Furthermore, the psychometric network models presented in the current project are visual representations of the positive manifold as interconnected networks of the interaction between pairs of cognitive tests, similar to how process overlap theory proposes formative *g* using the explanation of the positive manifold via overlapping general and specific processes. Ultimately, this demonstrates that psychometric network models are viable alternatives to latent variable models. In future, projects focused on theory building or theory assessment must first determine the underlying data-generating mechanisms assumed by the theories to establish whether a latent variable or psychometric network approach is most appropriate.

## 5. Limitations and Future Directions

The aim of this paper was to address specific comments by McFarland (2020) regarding the network models reported in Schmank et al. (2019). Therefore, many remaining important theoretical issues, as well as further analyses of the current data, are beyond the scope of the current paper. In particular, the conditions of comparability of the model fit of network and latent variable models will probably invoke important exchanges in the future.

A shortcoming of the current study is that it did not address the developmental question introduced by McFarland, that is, we did not repeat the analysis for different age categories. This clearly is a possible line of future research. In particular, such an analysis would make cross-validation possible, just like in the case of McFarland’s analysis.

Additionally, some of the models fitted by McFarland, such as a bifactor model and a penta-factor model, were not investigated in the current paper. Since these were important aspects of McFarland’s paper, further investigation on the matters discussed in this paper should address them. Our purpose with the current paper was to focus on what we perceived as central issues of McFarland’s paper as well as possible misconceptions of our previous results. There are excellent ideas and analyses in McFarland (2020) that have not been addressed here and which are worthy of exploration by future research.

## Figures and Tables

**Table 1:** Uncorrected correlation matrix provided by McFarland (2020): youngest group.

	BD	S	DS	MR	V	A	SS	VP	I	Cd	LN	FW	C	Ca	PC
BD	1.00														
S	0.43	1.00													
DS	0.39	0.34	1.00												
MR	0.52	0.37	0.42	1.00											
V	0.46	0.67	0.40	0.44	1.00										
A	0.47	0.41	0.60	0.48	0.51	1.00									
SS	0.36	0.30	0.27	0.31	0.29	0.33	1.00								
VP	0.66	0.42	0.34	0.51	0.47	0.47	0.32	1.00							
I	0.47	0.58	0.36	0.44	0.66	0.49	0.34	0.48	1.00						
Cd	0.32	0.29	0.34	0.35	0.28	0.43	0.57	0.30	0.29	1.00					
LN	0.42	0.34	0.67	0.35	0.39	0.52	0.27	0.35	0.40	0.22	1.00				
FW	0.54	0.48	0.46	0.51	0.51	0.58	0.29	0.55	0.49	0.32	0.42	1.00			
C	0.46	0.67	0.38	0.44	0.70	0.47	0.26	0.46	0.60	0.32	0.38	0.49	1.00		
Ca	0.38	0.24	0.44	0.30	0.30	0.41	0.42	0.37	0.34	0.39	0.33	0.31	0.24	1.00	
PC	0.47	0.44	0.37	0.39	0.38	0.36	0.29	0.47	0.42	0.24	0.35	0.40	0.46	0.31	1.00

Note. BD = block design; S = similarities; DS = digit span; MR = matrix reasoning; V = vocabulary; A = arithmetic; SS = symbol search; VP = visual puzzles; I = information; Cd = coding; LN = letter–number sequencing; FW = figure weights; C = comparisons; Ca = cancellation; PC = picture completion. *N* = 400.

**Table 2:** Uncorrected correlation matrix provided by McFarland (2020): middle group.

	BD	S	DS	MR	V	A	SS	VP	I	Cd	LN	FW	C	Ca	PC
BD	1.00														
S	0.47	1.00													
DS	0.44	0.49	1.00												
MR	0.54	0.52	0.49	1.00											
V	0.44	0.74	0.52	0.49	1.00										
A	0.51	0.55	0.61	0.52	0.59	1.00									
SS	0.39	0.33	0.41	0.36	0.35	0.38	1.00								
VP	0.67	0.48	0.43	0.54	0.43	0.49	0.35	1.00							
I	0.43	0.65	0.42	0.48	0.74	0.56	0.30	0.43	1.00						
Cd	0.35	0.35	0.43	0.42	0.38	0.43	0.64	0.33	0.29	1.00					
LN	0.43	0.46	0.71	0.46	0.50	0.59	0.36	0.43	0.44	0.37	1.00				
FW	0.56	0.54	0.52	0.58	0.53	0.61	0.33	0.59	0.51	0.34	0.52	1.00			
C	0.44	0.72	0.48	0.51	0.76	0.54	0.31	0.46	0.65	0.36	0.49	0.54	1.00		
Ca	0.32	0.19	0.32	0.25	0.20	0.28	0.46	0.31	0.18	0.42	0.28	0.26	0.17	1.00	
PC	0.49	0.36	0.36	0.39	0.34	0.31	0.39	0.47	0.33	0.31	0.35	0.39	0.33	0.33	1.00

Note. BD = block design; S = similarities; DS = digit span; MR = matrix reasoning; V = vocabulary; A = arithmetic; SS = symbol search; VP = visual puzzles; I = information; Cd = coding; LN = letter–number sequencing; FW = figure weights; C = comparisons; Ca = cancellation; PC = picture completion. *N* = 1000.

**Table 3:** Uncorrected correlation matrix provided by McFarland (2020): older group.

	BD	S	DS	MR	V	A	SS	VP	I	Cd	LN	FW	C	Ca	PC
BD	1.00														
S	0.52	1.00													
DS	0.44	0.52	1.00												
MR	0.57	0.50	0.52	1.00											
V	0.49	0.75	0.55	0.54	1.00										
A	0.54	0.63	0.58	0.55	0.64	1.00									
SS	0.48	0.38	0.44	0.47	0.35	0.39	1.00								
VP	0.64	0.43	0.38	0.51	0.46	0.52	0.45	1.00							
I	0.48	0.65	0.47	0.46	0.75	0.61	0.35	0.48	1.00						
Cd	0.44	0.47	0.48	0.51	0.43	0.46	0.65	0.39	0.36	1.00					
LN	0.40	0.51	0.65	0.51	0.51	0.53	0.48	0.43	0.44	0.54	1.00				
FW	0.58	0.57	0.51	0.60	0.58	0.64	0.43	0.59	0.53	0.46	0.47	1.00			
C	0.46	0.72	0.51	0.50	0.74	0.60	0.32	0.45	0.65	0.45	0.52	0.56	1.00		
Ca	0.37	0.36	0.31	0.26	0.29	0.32	0.48	0.32	0.23	0.46	0.37	0.36	0.32	1.00	
PC	0.55	0.49	0.41	0.45	0.49	0.45	0.44	0.54	0.51	0.42	0.43	0.49	0.51	0.36	1.00

Note. BD = block design; S = similarities; DS = digit span; MR = matrix reasoning; V = vocabulary; A = arithmetic; SS = symbol search; VP = visual puzzles; I = information; Cd = coding; LN = letter–number sequencing; FW = figure weights; C = comparisons; Ca = cancellation; PC = picture completion. *N* = 400.

**Table 4:** Model fit for latent variable and network models of WAIS-IV correlations (young group).

	Models	χ^2^	*df*	CFI/TLI	RMSEA	AIC	BIC
lavaan/qgraph	Correlated Factors	226.66 ***	84	0.95/0.94	0.07	298.66	328.12
	Higher-Order	232.22 ***	86	0.95/0.94	0.07	300.22	328.05
	Network	44.50	32	1.00/0.99	0.03	220.50	292.52
openMx	Correlated Factors	226.64 ***	84	0.95/0.94	0.07	298.64	328.10
	Network	44.48	32	1.00/0.99	0.03	220.48	292.50

Note. *** *p* < 0.001. χ^2^ = model chi-square value; *df* = degrees of freedom; CFI = Comparative Fit Index; TLI = Tucker–Lewis Index; RMSEA = root mean square error of approximation; AIC = Akaike information criteria; BIC = sample size adjusted Bayesian information criteria. AIC and BIC were calculated using the following formulas to allow for comparison across packages: AIC = χ^2^ + 2 × (120 − *df*); BIC = χ^2^ + ln(*N* + 2/24) × (120 − *df*).

**Table 5:** Model fit for latent variable and network models of WAIS-IV correlations (middle group).

	Models	χ^2^	*df*	CFI/TLI	RMSEA	AIC	BIC
lavaan/qgraph	Correlated Factors	469.08 ***	84	0.96/0.94	0.07	541.08	603.42
	Higher-Order	484.45 ***	86	0.95/0.94	0.07	552.45	580.27
	Network	50.50 *	32	1.00/0.99	0.02	226.50	378.89
openMx	Correlated Factors	469.08 ***	84	0.96/0.94	0.07	541.08	603.42
	Network	50.49 *	32	1.00/0.99	0.02	226.49	378.88

Note. * *p* < 0.05. *** *p* < 0.001. χ^2^ = model chi-square value; *df* = degrees of freedom; CFI = Comparative Fit Index; TLI = Tucker–Lewis Index; RMSEA = root mean square error of approximation; AIC = Akaike information criteria; BIC = sample size adjusted Bayesian information criteria. AIC and BIC were calculated using the following formulas to allow for comparison across packages: AIC = χ^2^ + 2 × (120 − *df*); BIC = χ^2^ + ln(*N* + 2/24) × (120 − *df*).

**Table 6:** Model fit for latent variable and network models of WAIS-IV correlations (older group).

	Models	χ^2^	*df*	CFI/TLI	RMSEA	AIC	BIC
lavaan/qgraph	Correlated Factors	245.63 ***	84	0.96/0.94	0.07	317.63	347.09
	Higher-Order	266.64 ***	86	0.95/0.94	0.07	334.64	362.47
	Network	27.28	30	1.00/1.00	<0.001	207.28	275.30
openMx	Correlated Factors	245.61 ***	84	0.96/0.94	0.07	317.61	347.07
	Network	27.26	30	1.00/1.00	<0.001	207.26	280.92

Note. *** *p* < 0.001. χ^2^ = model chi-square value; *df* = degrees of freedom; CFI = Comparative Fit Index; TLI = Tucker–Lewis Index; RMSEA = root mean square error of approximation; AIC = Akaike information criteria; BIC = sample size adjusted Bayesian information criteria. AIC and BIC were calculated using the following formulas to allow for comparison across packages: AIC = χ^2^ + 2 × (120 − *df*); BIC = χ^2^ + ln(*N* + 2/24) × (120 − *df*).

## Data Availability

The data and statistical analyses presented in this study are openly available via the Open Science Framework at https://doi.org/10.17605/OSF.IO/KFUJS.

## Footnotes

1. The present paper and Schmank et al. (2019) employ RMSEA cutoff values representing the strictest value deemed acceptable by Schreiber et al. (2006); it should be noted that values less than 0.08 can also indicate adequate model fit (Browne and Cudeck 1993; Schermelleh-Engel et al. 2003).
